# Incomplete punctal canalization -a balloon variant of the external membrane: a case report

**DOI:** 10.1186/1752-1947-8-120

**Published:** 2014-04-09

**Authors:** Mohammad Javed Ali, Milind N Naik

**Affiliations:** 1 Dacryology Service, Ophthalmic Plastics Surgery, L.V. Prasad Eye Institute, Road No 2, Banjara Hills, Hyderabad 500034, India

**Keywords:** Incomplete punctal canalization, Membranotomy, Punctum, Balloon

## Abstract

**Introduction:**

Incomplete punctal canalization is an uncommon congenital disorder characterized by a dysgenetic punctum with membranes. External membranes, the most common type of incomplete punctal canalization are flat and overlie the punctum as a veil. We describe a newer variant of incomplete punctal canalization, its clinical profile, diagnostic criteria and management.

**Case presentation:**

A 9-year-old Indian boy presented with watering of his right eye since birth. His right eye lower punctal area showed an avascular translucent elevation that appeared to have a smooth dome shape. An examination at high magnification showed the slopes of the dome gradually merging and contiguous with the tarsal conjunctiva. Based on a very high degree of suspicion, an impression of atypical external membrane variety of incomplete punctal canalization was made. Membranotomy was successful in the management of his condition.

**Conclusions:**

A high degree of suspicion is the key point in the diagnosis of this variant, keeping in mind the other features described for incomplete punctal canalization- external membrane. It is possible that ballooning of these membranes may represent an evolutionary stage in the process of complete canalization and this could be the starting point for further dacryo-embryologic exploration and correlations.

## Introduction

Proximal lacrimal outflow dysgenesis involving the punctum and canaliculus is sparsely documented in the literature [1-6]. The term incomplete punctal canalization (IPC) was first introduced by Ali *et al.*[7]. They described the largest series of IPC and classified it into an external membrane variety (IPC-EM) and one with an internal membrane (IPC-IM) [7]. We report the clinical and diagnostic profile and management of a balloon variant of the external membrane along with the clincopathological correlation and propose that this variant be included in the subclassification of IPC-EM.

## Case presentation

A 9-year-old healthy Indian boy presented with watering of his right eye since birth that was not associated with any discharge or swelling at the corner of the eye. There were no complaints in the left eye. His best corrected visual acuity (BCVA) was 20/20 in both eyes and the rest of his ocular examination was normal.

**Figure 1:**
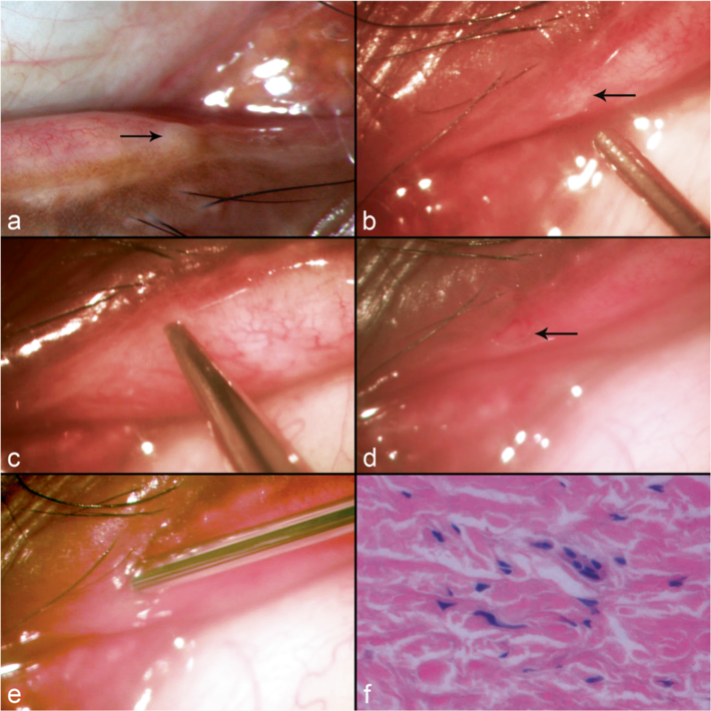
**Clinical profile of balloon variant of incomplete punctal canalization external membrane variety.** Slit lamp photograph of the right lower lid showing an avascular, elevated and translucent membrane covering the puncta (arrow) **(a)**. Intraoperative photograph of the same patient showing the dome-shaped elevation with slopes contiguous with the tarsal conjunctiva (arrow) **(b)**. Intraoperative photograph showing the process of membranotomy with a Nettleship’s punctum dilator **(c)**. Intraoperative photograph following membranotomy. Note the wide punctal opening (arrow) **(d)**. Intraoperative photograph showing a probe in the normal canaliculus **(e)**. Microphotograph of the membrane showing fibrocollagenous tissue with few vascular channels without any evidence of inflammation suggestive of fibrovascular membranes (hematoxylin and eosin x400) **(f)**.

On examination, the lids appeared normal but the tear meniscus in his right eye was found to be elevated. An examination of the medial aspect of his upper lid revealed an agenetic punctum without any evident signs of IPC. However, the lower punctum area showed an avascular translucent elevation that appeared to have a smooth dome shape (Figure 1a). Examination at high magnification showed that the slopes of the dome gradually merged and were contiguous with the tarsal conjunctiva (Figure 1b). The architecture of the pars lacrimalis portion of his lid was normal. Based on a very high degree of suspicion, an impression of atypical IPC was made.

Our patient underwent membranotomy with a gradual tapered Nettleship’s punctum dilator (Figure 1c) as described earlier [7]. Following membranotomy, the punctum was well seen with a good opening (Figure 1d). Probing revealed the lower canaliculus to be normal (Figure 1e) and the nasolacrimal passage was found to be patent on irrigation. The lacrimal system of the fellow eye was normal. At the three-month follow-up, the right lower punctum was found to be normal with grade 1 dye disappearance and our patient reported resolution of his epiphora.

A histopathological examination of the membrane was consistent with that of the IPC membranes reported in the literature [7]. It showed a fibrocollagenous tissue with few vascular channels without any evidence of inflammation suggestive of fibrovascular membranes (Figure 1f).

## Discussion

The lacrimal passages develop along the line of the cleft between the lateral nasal process and the maxillary process of the embryonic face [6,7]. The process of canalization begins in a 35mm embryo by apoptosis of the central cells. The entire canaliculus is canalized except near the puncta and this portion opens onto the lid surface in a 130mm embryo, before the separation of the eyelids at the seventh month of intrauterine life [3,4,6,8].

The pathogenesis of punctal membranes is unknown but is believed to be due to failure of canalization of the most proximal part of lacrimal apparatus [1,3,7,8]. Incomplete punctal canalization thus represents the mildest form of proximal lacrimal outflow dysgenesis [1,7].

The IPC-EM type is described in the literature to typically cover the external surface of the puncta like a flat translucent veil in an area of an avascular dimple [7]. However in the present case, although there was an avascular and translucent area, the dimple was characteristically absent. Instead, there was a dome-shaped elevation as elucidated in the case report. Additional help in the diagnosis was the normal architecture of the pars lacrimalis of the lower lid. There was no systemic association in the present case although there was an associated lacrimal anomaly in the form of upper punctal agenesis.

Membranotomy was found to be a useful modality of management for IPC with a functional success of 91% [7]. Membranotomy in the present case was patent at the last follow-up with resolution of the epiphora. The histopathological examination was consistent with IPC membranes as reported in the literature [7].

## Conclusions

In conclusion, a high degree of suspicion is needed for the diagnosis of this variant keeping in mind the other features described for IPC-EM. It is possible that ballooning of these membranes may represent an evolutionary stage in the process of complete canalization and this could be the starting point for further dacryo-embryologic exploration and correlations.

## Consent

Written informed consent was obtained from the patient’s legal guardian for publication of this case report and any accompanying images. A copy of the written consent is available for review by the Editor-in-Chief of this journal.

## Abbreviations

BCVA: best corrected visual acuity; IPC: incomplete punctal canalization: a congenital membranous disorder of the lacrimal punctum; IPC-EM: incomplete punctal canalization- external membrane; IPC-IM: incomplete punctal canalization -internal membrane.

## Competing interests

The authors declare that they have no competing interests.

## Authors’ contribution

AMJ conceived of the study and drafted the manuscript. NMN helped with the drafting and critical review of the manuscript. Both authors read and approved the final manuscript.
